# Inkjet Printing-Based Immobilization Method for a Single-Step and Homogeneous Competitive Immunoassay in Microchannel Arrays

**DOI:** 10.3389/fchem.2020.612132

**Published:** 2020-12-21

**Authors:** Yuko Kawai, Akihiro Shirai, Masaya Kakuta, Kotaro Idegami, Kenji Sueyoshi, Tatsuro Endo, Hideaki Hisamoto

**Affiliations:** ^1^Department of Applied Chemistry, Graduate School of Engineering, Osaka Prefecture University, Osaka, Japan; ^2^Sysmex Corporation, Hyogo, Japan

**Keywords:** C-reactive protein, immunoassay, inkjet printing, poly(dimethylsiloxane) (PDMS), microchannel, single-step

## Abstract

In this study, we report an inkjet printing-based method for the immobilization of different reactive analytical reagents on a single microchannel for a single-step and homogeneous solution-based competitive immunoassay. The immunoassay microdevice is composed of a poly(dimethylsiloxane) microchannel that is patterned using inkjet printing by two types of reactive reagents as dissolvable spots, namely, antibody-immobilized graphene oxide and a fluorescently labeled antigen. Since nanoliter-sized droplets of the reagents could be accurately and position-selectively spotted on the microchannel, different reactive reagents were simultaneously immobilized onto the same microchannel, which was difficult to achieve in previously reported capillary-based single-step bioassay devices. In the present study, the positions of the reagent spots and amount of reagent matrix were investigated to demonstrate the stable and reproducible immobilization and a uniform dissolution. Finally, a preliminary application to a single-step immunoassay of C-reactive protein was demonstrated as a proof of concept.

## Introduction

In the field of bioassays, microplates are widely used as analytical tools. However, it is well known that bioassay methods, including immunoassay or enzyme assays using microplates, require complicated operational procedures and large sample volumes (Ng et al., 2010). Recently, paper-based analytical devices (μPADs) have received significant attention owing to their simplicity, low cost, and low sample volume (Martinez et al., 2010; Komuro et al., 2013; Yetisen et al., 2013; Hu et al., 2014; Cate et al., 2015; He et al., 2015; Xia et al., 2016). Colorimetric assay is standard for PADs but electrochemiluminescence assay is also used (Feng et al., 2014; Sun et al., 2018). Excellent results have been reported in many studies; however, PADs can still undergo reagent bleeding and subsequent inhomogeneous color changes. In the field of microfluidic immunoassays, capillary electrophoresis-based immunoassays have been investigated over the past few decades (Koutny et al., 1996; Von Heeren et al., 1996; Chiem and Harrison, 1997; Cheng et al., 2001; Kawabata et al., 2005). These immunoassays are based on the electrophoretic separation of antigen-antibody complexes and their free forms. Thus, rapid and efficient separation and detection can be achieved by capillary electrophoresis. Microfluidic immunosorbent assays, such as enzyme-linked immunosorbent assay (ELISA), have also been reported (Sato et al., 2000, 2001; Wang et al., 2012; Apilux et al., 2013; Furutani et al., 2018; Khodayari Bavil and Kim, 2018). Immunosorbent assays are based on the immobilization of antigens or antibodies on a solid surface to achieve a high sensitivity and selectivity. However, these systems require complicated operational procedures such as the application of an electric field and step-by-step reactions. Although traditional lateral flow assays have undergone a number of improvements in recent years (Nishat et al., 2019; Zhan et al., 2019; Alhussien and Dang, 2020; Lee et al., 2020; Wu et al., 2020), they tend to limit multiplex detection. Lateral flow assays enabling multiplex detection was reported (Engels et al., 2018), however, this assay requires washing step. On the other hand, our group recently developed reagent-release capillary-based single-step bioassay microdevices using glass capillaries or poly(dimethylsiloxane) (PDMS) microchannels. This approach allows the simple fabrication of multi-sensing devices by simply arraying various single-step sensing capillaries (Henares et al., 2007, 2008; Fujii et al., 2012; Uchiyama et al., 2012). Thus, the simultaneous detection of different chemical species can be achieved through the simple introduction of a sample solution by capillary action. In addition, single-step enzyme inhibitor assays or competitive bioassay devices have been fabricated by combining two PDMS microchannel arrays independently coated with different reactive reagents, enzymes and fluorescent substrates, or biotin-coated graphene oxide and fluorescent avidin to achieve a single-step assay (Uchiyama et al., 2012; Ishimoto et al., 2013, 2014; Shirai et al., 2017). However, the fabrication of these microdevices remains problematic due to the fact that two reactive reagents are inhomogeneously immobilized, and the alignment of two PDMS microchannel arrays without a gap is technically complicated.

**Figure 1:**
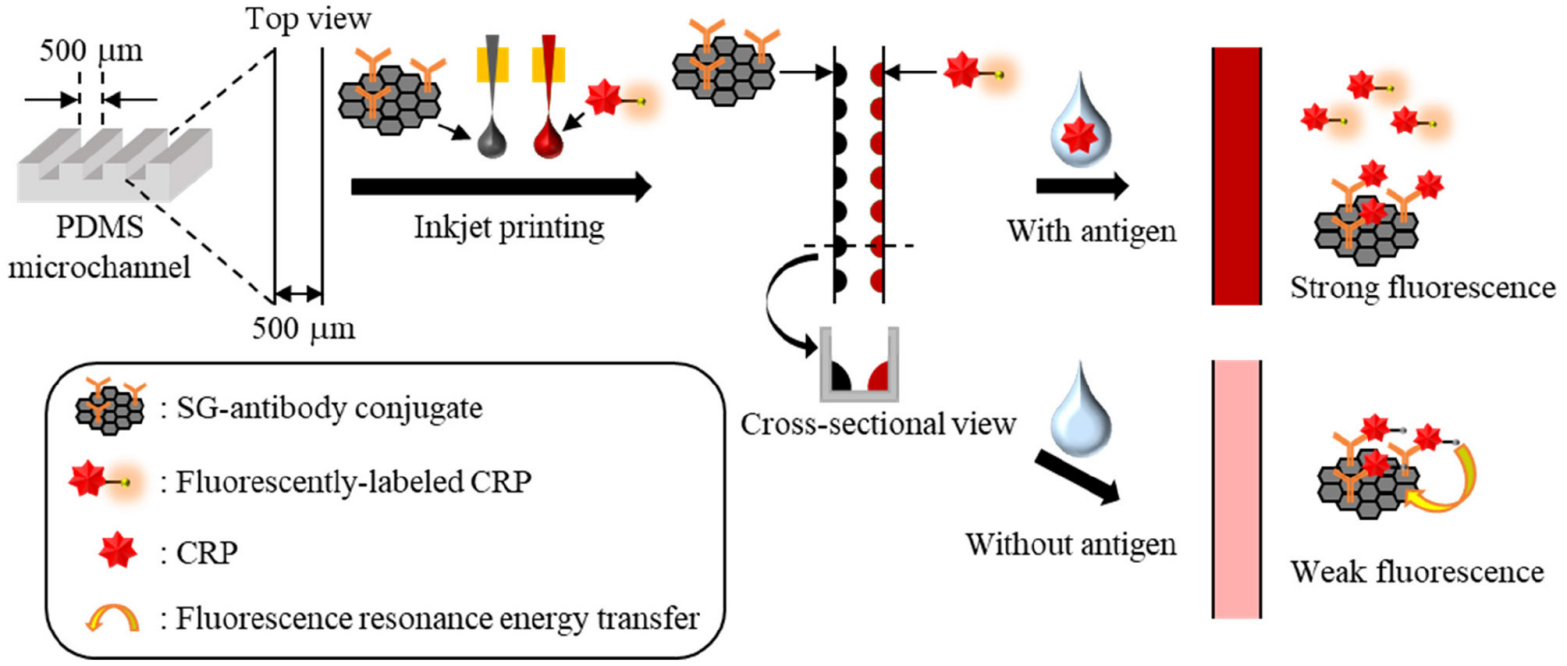
Immunoassay principle of a single-step CRP competitive immunoassay microdevice based on inkjet printing.

Thus, we herein propose a new method for the immobilization of two reactive reagents onto a single microdevice for the simple fabrication of single-step and homogeneous solution-based bioassay devices using inkjet printing. These reactive reagents are separately immobilized as reagent spots on the two bottom corners of a single microchannel. This fabrication method makes the aligning procedure unnecessary. Moreover, the inkjet printer can accurately and homogeneously dispense reagents as nanoliter-droplets onto a microchannel, which leads to a reliable and mass-producible fabrication of bioassay devices. To demonstrate the applicability of this method, a single-step competitive immunoassay microdevice for C-reactive protein (CRP) is prepared by the immobilization of a sulfonic acid group-containing graphene (SG)-antibody conjugate and a fluorescently labeled CRP (Figure 1).

In the presence of CRP in the sample solution, CRP and the fluorescently labeled CRP bind SG- antibody conjugate competitively; thus, the fluorescence of the free fluorescently labeled CRP can be measured. On the other hand, in the absence of CRP in the sample solution, the fluorescently labeled CRP is fully bound with the SG-antibody conjugate, and therefore, the fluorescence of the fluorescently labeled CRP is quenched by fluorescence resonance energy transfer (FRET) on the SG. Here, an inkjet printer is employed to accurately and position-selectively immobilize two reactive reagents in the same microchannel to demonstrate a single-step and homogeneous solution-based competitive immunoassay as an example.

## Materials and Methods

### Materials

The PDMS pre-polymer (SILPOT 184) and curing agent (SILPOT184 CAT) were purchased from Dow Corning Toray Co., Ltd. (Tokyo, Japan). Anti-CRP antibody (C5) was purchased from Abcam (Cambridge, UK). Recombinant human CRP was purchased from ORIENTAL YEAST Co., Ltd. (Tokyo, Japan). LK14 HiLyte Fluor™ 555 Labeling Kit—NH_2_ was purchased from Dojindo (Kumamoto, Japan). Tween 20 and sodium carbonate were purchased from Tokyo Chemical Industry Co., Ltd. (Tokyo, Japan). Endotoxin-free trehalose was purchased from HAYASHIBARA Co., Ltd. (Okayama, Japan). RNase-free Tris (1 M, pH 8.0) was purchased from Thermo Fisher Scientific Inc. (Massachusetts, USA). Graphene oxide nanocolloids (2 mg/mL dispersion in H_2_O), poly [dimethylsiloxane-co-methyl(3-hydroxypropyl)siloxane]-graft-poly(ethylene glycol) methyl ether (PDMS-PEG) and hemoglobin human (Hb) lyophilized powder were purchased from Sigma-Aldrich (Tokyo, Japan). Hydrochloric acid and human serum albumin (HSA) were purchased from Wako Pure Chemical Industries (Osaka, Japan). The deionized water employed throughout exhibited a resistivity value >1.8 × 10^7^ Ω cm at 25°C, which was prepared by ultra-pure water purification system (Merck Direct-Q-UV3).

### Preparation of the SG-Antibody Conjugate Solution for Inkjet Printing

To increase the solubility of graphene oxide in water, sulfonic acid-containing graphene oxide (SG) was synthesized from graphene oxide using a previously reported method (Si and Samulski, 2008; Shirai et al., 2016). To prepare the SG-antibody conjugates, 500 μL of aqueous solution containing SG (200 μg mL^−1^) and anti-CRP antibody (80 μg mL^−1^) were prepared and incubated at 4 °C overnight. After this time, 10 μL of Tween 20 in water (10 vol%) was added as a blocking reagent. After 2 h, the resulting mixture was subjected to centrifugation for 20 min at 15,000 rpm and rinsed with water. This step was repeated twice. Trehalose (160 μg mL^−1^), which was used as the immobilization matrix and has a high solubility in water, was added to give the SG-antibody conjugate (SG: 800 μg mL^−1^) containing 16% (w/v) trehalose for use in inkjet printing.

### Preparation of the Fluorescently Labeled CRP Solution for Inkjet Printing

To prepare the fluorescently labeled CRP, anti-CRP antibody (500 μg mL^−1^) was bound with HiLyte Fluor™ 555 using LK14 HiLyte Fluor™ 555 Labeling Kit—NH_2_. Subsequently, fluorescently labeled CRP was diluted with water and trehalose (80 μg mL^−1^) was added to give the fluorescently labeled CRP (4 μg mL^−1^) containing 8% (w/v) trehalose for use in inkjet printing.

### Fabrication of the PDMS Microchannel Arrays

For preparation of the immunoassay microdevice, microchannel arrays (channel width: 500 μm, channel depth: 500 μm) were fabricated on PDMS by a molding operation from a glass mold. More specifically, the PDMS prepolymer was introduced onto a glass mold (channel width: 500 μm, channel depth: 500 μm), dried under vacuum for 1.5 h, and heated for 1.5 h at 70°C. Subsequently, a PDMS microchannel array plate was peeled off from the glass mold to give the first PDMS mold. These operations were repeated again using the first PDMS mold instead of the glass mold, and the PDMS microchannel array plate was peeled off from the first PDMS mold, cut into 1 cm long pieces, and used for further experiments.

### Fabrication of the Immunoassay Microdevice

For preparation of the immunoassay microdevice, two reactive reagents containing trehalose (i.e., the SG-antibody conjugate and fluorescently labeled CRP) were separately spotted as droplets (30 nL each) at the two bottom corners of a PDMS microchannel by inkjet printing (Microjet, Labojet-1000P, Nagano, Japan). The minimum volume and corresponding spot diameter of a droplet produced using the inkjet printer employed herein were ~15 nL and 300 μm, respectively. Since the above-prepared spotting solution is highly viscose, a further increase in the concentration of the solution for use in inkjet printing is difficult. Therefore, the amount of immobilizing reagents was adjusted by varying the number of spots, whereby two droplets (30 nL) were spotted. The final concentration of the SG-antibody conjugate after sample introduction was 100 μg mL^−1^ (containing 2% (w/v) trehalose), and that of the fluorescently labeled CRP was 0.5 μg mL^−1^ (containing 1% (w/v) trehalose). The PDMS microchannel arrays immobilizing the two reactive reagents were dried at 15°C for 1–3 d. During the fluorescence measurements, a cover glass was placed on the microchannel arrays to prevent drying of the CRP-containing sample solutions.

### Single-Step Competitive Immunoassay Using the Microdevice

The sample solutions containing CRP at concentrations ranging from 0 to 50 μg mL^−1^ were introduced into the immunoassay microdevice by capillary action, and both ends of the microchannel were sealed using a PDMS prepolymer to prevent drying. Subsequently, fluorescence measurements were carried out for the various CRP concentrations using a fluorescence microscope equipped with a CCD camera (Keyence, VB-7010, Osaka, Japan) after 1 h of incubation.

## Results and Discussion

**Figure 2:**
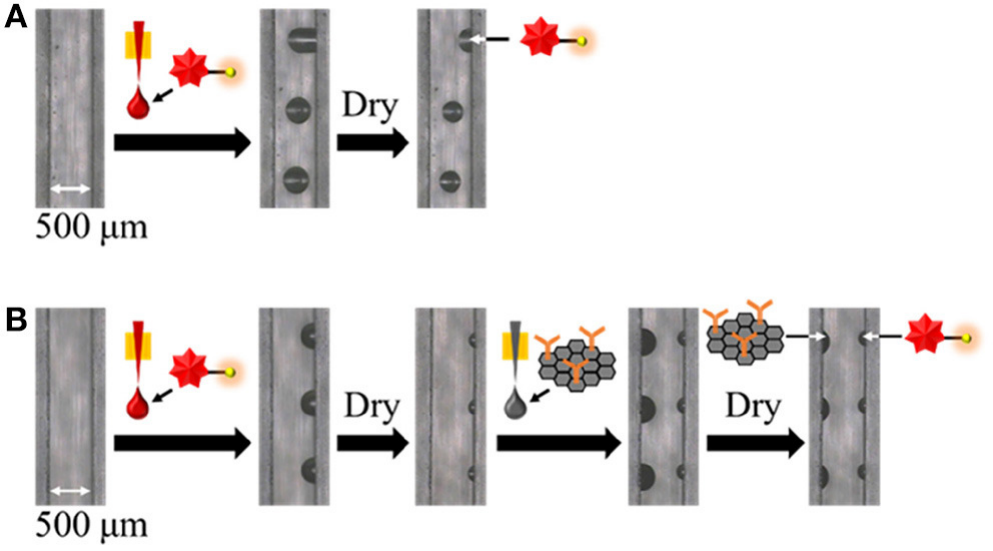
Investigation of the immobilizing positions of two reactive reagents as reagent spots at **(A)** the bottom center, and **(B)** the two bottom corners.

Initially, to confirm the attachment of antibody on SG surface, fourier transform infrared spectroscopy (FTIR) measurements were performed. Supplementary Figure 1 shows FTIR transmittance spectra of SG and SG-antibody conjugates. In the case of SG-antibody conjugates, the peak of C=O stretching vibration of amide group was clearly observed at 1648 cm^−1^, on the other hand, that was not observed in the case of SG alone. Thus, attachment of antibody on SG surface was confirmed. Then, the immobilizing positions of the two reactive reagents (i.e., the SG-antibody conjugate and the fluorescently labeled CRP) as reagent spots were investigated. The distance between the inkjet printer (i.e., the capillary tip) and the channel bottom surface was set to 1,500 μm (1,000 μm to the top PDMS surface) to ensure stable spotting. In this case, the minimum spot size on the PDMS surface was ~300 μm. Since the PDMS channel size was ~500 μm, we initially spotted the reagents at the center of the microchannel along the longitudinal direction (Figure 2A). In this case, spotted droplets often moved and came into contact with the channel wall during the spotting process because of difficulties in achieving complete alignment (Figure 2A). However, when the spotting position was shifted to the beside the channel wall (i.e., 100–150 μm from the channel wall), we found that the spotted droplets were reproducibly fixed at the bottom corners of the microchannel (Figure 2B). This may be caused by a stronger adhesion force at the bottom corners compared to that at the flat part of the channel bottom. In addition, fixing at the bottom corners gave smaller droplet sizes, which rendered the immobilization of two different reagent spots on both sides of the channel bottom more facile (Figure 2B).

**Figure 3:**
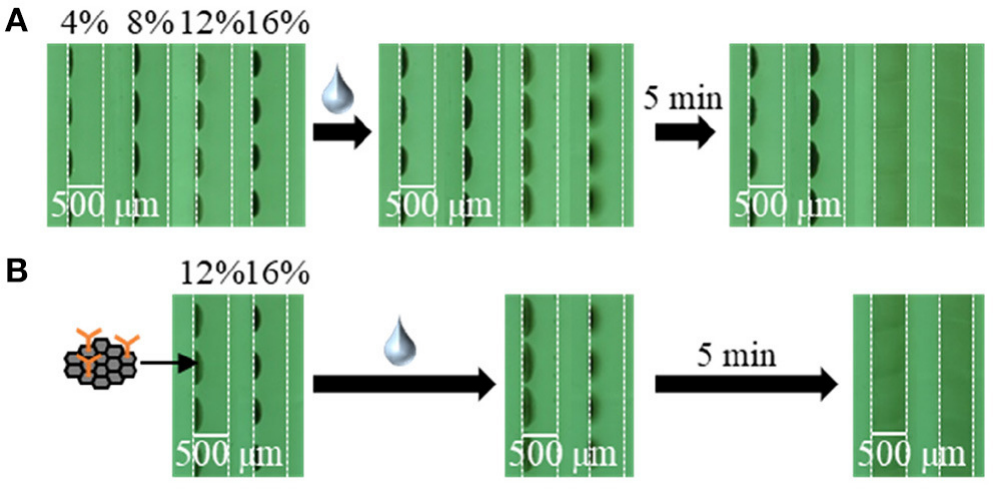
Bright field images of SG-antibody conjugate spots containing 4–16% trehalose after drying **(A)** for 1 h, and **(B)** overnight, and subsequent dissolution by sample introduction.

**Figure 4:**
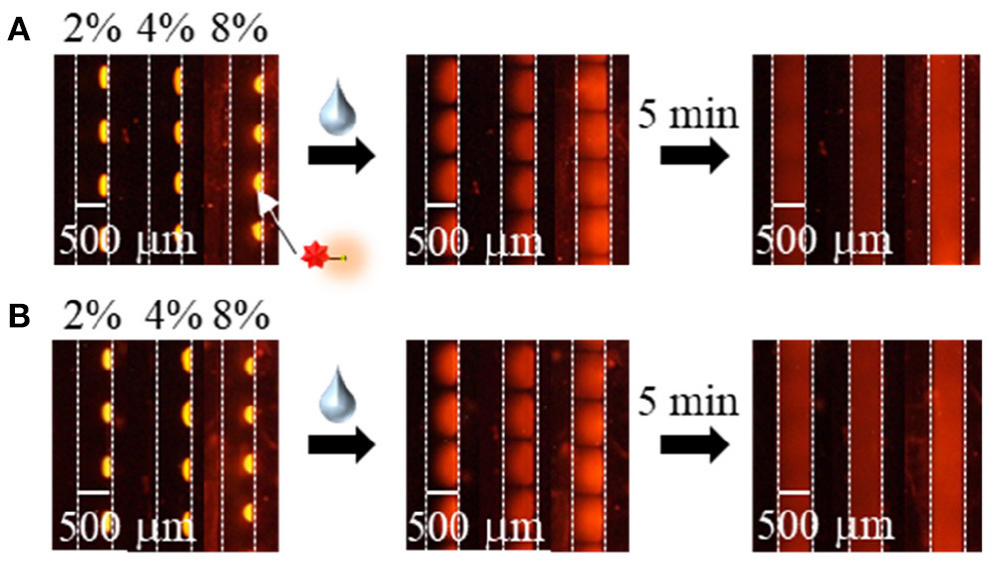
Fluorescence images of the fluorescently labeled CRP spots containing 2–8% trehalose after drying **(A)** for 2–4 h, and **(B)** overnight, and subsequent dissolution by sample introduction.

To apply the inkjet-based immobilization process to a homogeneous solution-based single-step competitive immunoassay in a microchannel, the complete dissolution of two reactive reagents within a reasonable amount of time by sample introduction is essential. Thus, we carried out preliminary experiments using BSA and trehalose as typical immobilization matrices and found that trehalose was suitable for dissolution (Kawai et al., in press); therefore, the amount of trehalose required in the reagent solution for inkjet-based immobilization was investigated (see Figures 3, 4). In terms of the dissolution of the SG-antibody conjugates, an immobilization solution containing 16% trehalose resulted in full dissolution within 5 min (Figure 3). In addition, investigation of the drying time for the reagent spots showed that no change in the dissolution behavior was observed between 1 h and overnight drying (Figures 3A,B). On the other hand, in the case of the fluorescently labeled CRP solutions containing different amounts of trehalose (i.e., 2, 4, and 8%), homogeneous dissolution was observed in all tested conditions (Figure 4). However, when the fluorescently labeled CRP solution containing 8% trehalose was used, the fluorescence intensity was brighter than in the cases of the solutions containing 2 and 4% trehalose. This may be caused by solubility differences in the fluorescently labeled CRP, i.e., higher concentration of trehalose ensured the dissolution of the immobilized reagent. Based on these results, an SG-antibody conjugate solution containing 16% trehalose and a fluorescently labeled CRP solution containing 8% trehalose were determined to be the optimal concentrations allowing complete dissolution of these reagents for sample spotting.

**Figure 5:**
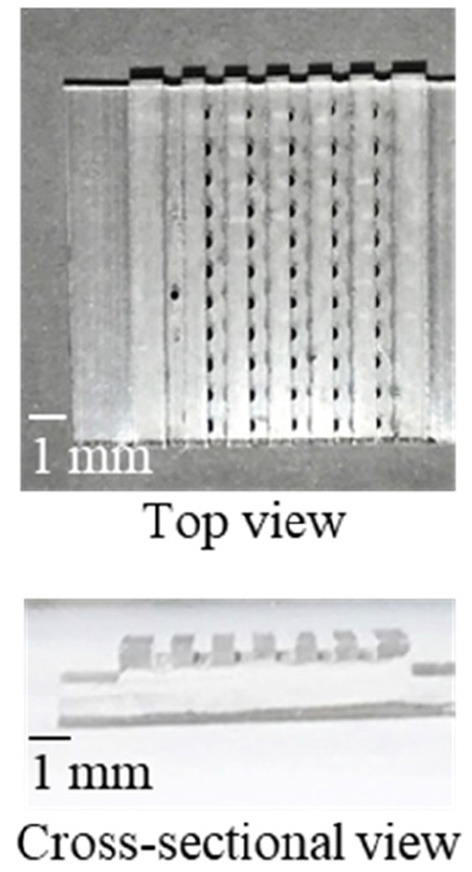
The fabricated microdevice. The optically visible black dots are the SG-antibody conjugate spots.

**Figure 6:**
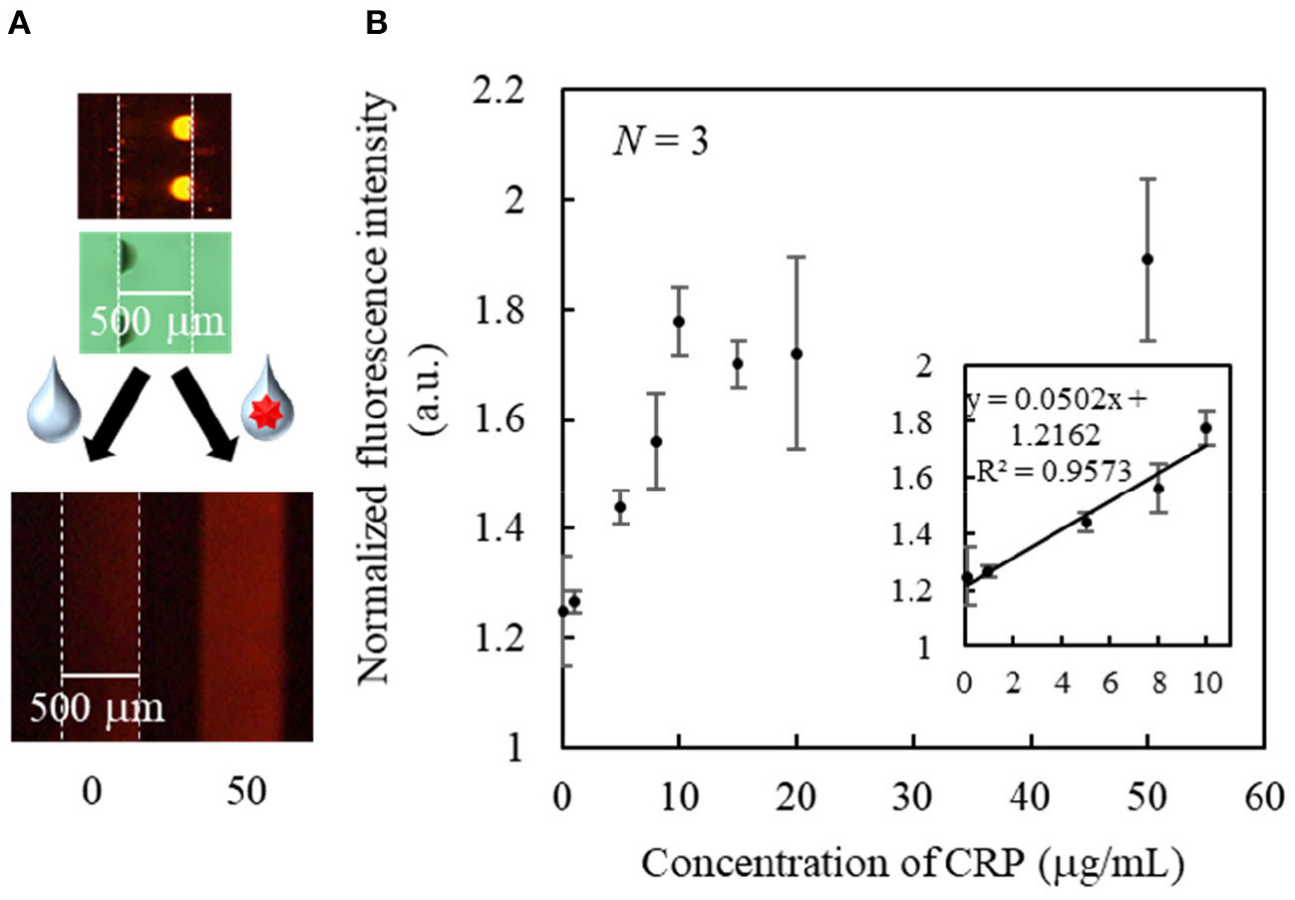
**(A)** Bright field and fluorescence images of the immobilized spots, and fluorescence images of typical responses after sample introduction. **(B)** Standard curve for the single-step CRP immunoassay.

As a proof of concept, a single-step and homogeneous competitive immunoassay microdevice was fabricated by spotting the two reagents of the optimized concentrations by inkjet printing onto the two bottom corners of the microchannel. Figure 5 shows the top and cross-sectional views of the device after spotting the two reagents on both sides of the channel bottom. As shown in Figure 5, although the fluorescently labeled CRP was not visible, periodic black spots of the SG-antibody were clearly observed on the left-hand side of the microchannel, confirming their accurate immobilization. Figure 6A shows an image of the two reactive reagents immobilized on the microdevice. More specifically, the SG-antibody conjugate containing 16% trehalose was only observed in the bright-field image, while the fluorescently labeled CRP containing 8% trehalose was only observed in the fluorescence image. Figure 6A clearly shows that the two reactive reagents were successfully immobilized as spots at different positions on the single microchannel using our inkjet printing technique. Furthermore, when the buffer solution was introduced into the channel, the fluorescence was quenched; on the other hand, introduction of the CRP sample solution resulted in a fluorescence response. Thus, the initial concept was successfully achieved. Figure 6B shows the normalized fluorescence intensity of the microchannel after introduction of the CRP sample solution into the microdevice. This normalized intensity was calculated by dividing the fluorescence intensity obtained from the CRP-containing sample solution by that obtained from the blank buffer solution. As expected, the normalized fluorescence intensity increased upon increasing the CRP concentration (0.1–10 μg mL^−1^) in the sample solution. From these results, the detection of CRP was successfully performed using a single-step operation. The limit of detection (LOD, i.e., 3δ of the blank signal) was calculated to be 2.5 μg mL^−1^. Table 1 shows the comparison of detection range, LOD, and operation procedure of typical CRP biosensors reported in previous reports. Cut-off value of CRP in blood sample is typically 3 μg mL^−1^, and higher concentration causes high risk of diseases (Ridker, 2003). LODs of other biosensors were better than those of our report. ELISA (Vashist et al., 2015) and paper-based microfluidic assay (Dong et al., 2017) required multi operation steps, then lateral flow immunoassay (Cai et al., 2018) needed complicated device fabrication. Electroosmotic lateral flow immunoassay (Oyama et al., 2012) also required multi operation steps and electrophoretic separation of complexes and free forms. Compared to these reports, our immunoassay microdevice covered the concentration ranges including the cut-off value, and operation procedure is also single step. Furthermore, fabrication process using inkjet printing is also quite simple and advantageous.

**Table 1:** Comparison of CRP biosensors.

**Assay type**	**Detection range**	**LOD**	**Operation**	**Reference**
Lateral flow immunoassay	0.1–160 mg/L	0.091 mg L^−1^	Single-step	Cai et al., 2018
Electroosmotic lateral flow immunoassay	8.5 ng mL^−1^−5 μg mL^−1^	8.5 ng mL^−1^	Multi-step	Oyama et al., 2012
ELISA	0.03–81 ng mL^−1^	0.07 ng mL^−1^	Multi-step	Vashist et al., 2015
Paper-Based Microfluidic Immunoassay	Up to 2 μg mL^−1^	54 ng mL^−1^	Multi-step	Dong et al., 2017
Homogeneous immunoassay	2.5–10 μg mL^−1^	2.5 μg mL^−1^	Single-step	Our method

Then, the storage stability of the immunoassay microdevice was evaluated. Supplementary Figure 2 shows the fluorescence intensities of the microchannels when the devices were stored at 15 degree Celsius for 1, 5, 6, or 7 days and CRP (50 μg mL^−1^) sample solutions were introduced. The fluorescence intensity remained constant after 7 days storage. Therefore, our device has at least 7 days storage stability.

**Figure 7:**
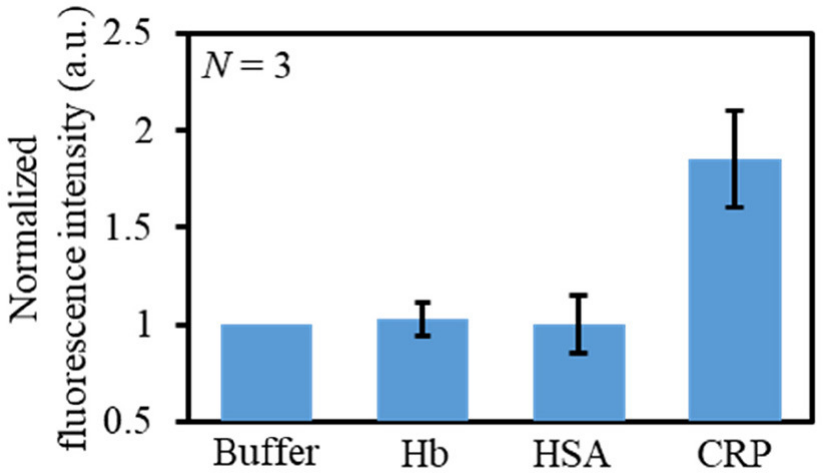
Evaluation of the selectivity of the immunoassay microdevice using different solutions: buffer, HB (50 μg mL^−1^), HSA (50 μg mL^−1^), and CRP (50 μg mL^−1^).

Finally, the selectivity of this immunoassay microdevice toward CRP was investigated using typically coexisting proteins, namely Hb and HSA. Figure 7 shows the fluorescence responses toward 50 μg mL^−1^ solution of Hb, HSA, and CRP, whereby it is apparent that the fluorescence intensity was higher in the case of CRP than for Hb and HSA. Moreover, the fluorescence intensities of Hb and HSA were comparable to those of the blank buffer solution. These results therefore confirm the CRP selectivity of the developed immunoassay microdevice.

## Conclusions

In conclusion, here, we reported the use of inkjet printing to pattern two reactive reagents at different positions of the same microchannel. More specifically, the immunoassay microdevice described herein is composed of a PDMS microchannel that is patterned using antibody-immobilized graphene oxide and a fluorescently labeled antigen as the above-mentioned reactive reagents. We found that spotting at the bottom corners of the microchannel allowed reproducible and position-selective immobilization of the reagents, and the use of trehalose as an immobilization matrix allowed the successful dissolution of the immobilized reagents. Furthermore, the fabrication of a single-step and homogeneous competitive immunoassay microdevice by inkjet printing was successfully achieved, and the detection of CRP was demonstrated as a proof of concept. Although sensing performance of present CRP detection still needs improvement, inkjet printing allows the accurate and position-selective immobilization of various reagents, thus, the present work is expected to widen the application of inkjet printing to various microchannel-based devices.

## Data Availability Statement

The raw data supporting the conclusions of this article will be made available by the authors, without undue reservation.

## Author Contributions

HH, MK and KI conceived of the idea to use inkjet printing for immobilization. All the experiments were carried out by YK and AS. KS, TE and HH contributed to the data analysis. Preparation of immobilizing reagents were established by AS. The initial paper draft was written by YK and HH, with editorial input and final editing by KS and TE. The final draft was prepared and uploaded by HH.

## Conflict of Interest

The authors declare that this study received funding from Sysmex Corporation, the Uehara Memorial Foundation, and the Japan Society for Promotion of Science (20H02770). The funders were not involved in the study design, collection, analysis, interpretation of data, the writing of this article or the decision to submit it for publication.
