# Supplementary material for: Inkjet Printing-Based Immobilization Method for a Single-Step and Homogeneous Competitive Immunoassay in Microchannel Arrays
Source: Front Chem. 2020 Dec 21;8:612132. doi: 10.3389/fchem.2020.612132 (PMC7779625; doi:10.3389/fchem.2020.612132)
Supplement: Supplementary file 1 [file Data_Sheet_1.docx]

Supplementary Material

Inkjet Printing-based Immobilization Method for a Single-step and Homogeneous Competitive Immunoassay in Microchannel Arrays

Yuko Kawai^1^, Akihiro Shirai^1^, Masaya Kakuta^2^, Kotaro Idegami^2^, Kenji Sueyoshi^1^, Tatsuro Endo^1^, Hideaki Hisamoto^1^*

^1^Department of Applied Chemistry, Graduate School of Engineering, Osaka Prefecture University, Osaka, Japan

^2^Sysmex Corporation, Hyogo, Japan

*** Correspondence:**Hideaki Hisamoto
hisamoto@chem.osakafu-u.ac.jp


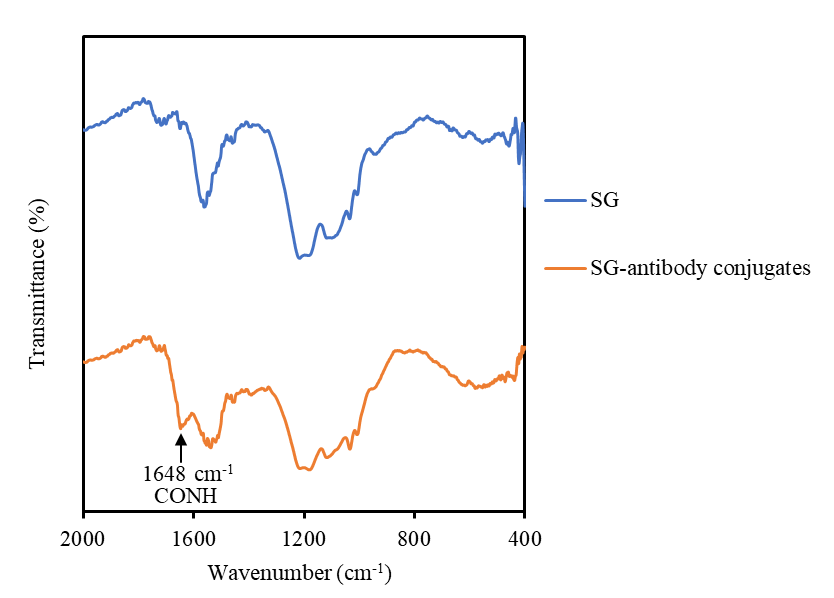


**Figure S1.** FTIR transmittance spectra of SG and SG-antibody conjugates. Appropriate amount of SG or SG-antibody powder was sandwiched by two KBr disks to form KBr pellet by compression. Transmittance spectra were measured by FT/IR 4200 (JASCO, Tokyo, Japan).


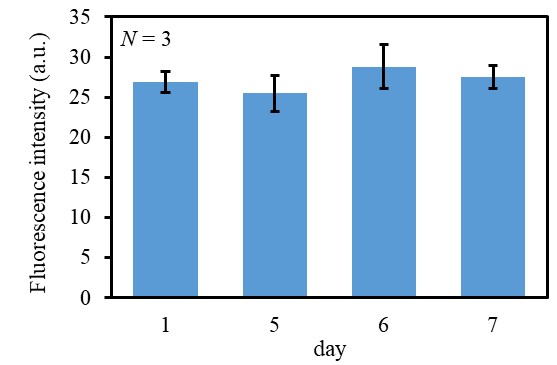


**Figure S2.** Evaluation of the storage stability of the immunoassay microdevice at room temperature. Fluorescence responses were obtained by using CRP (50 μg mL^−1^) sample solutions.
